# *Plasmodium knowlesi* infection in a returning German traveller from Thailand: a case report on an emerging malaria pathogen in a popular low-risk travel destination

**DOI:** 10.1186/s12879-018-3059-z

**Published:** 2018-04-02

**Authors:** Guenter Froeschl, Marcus Beissner, Kristina Huber, Gisela Bretzel, Michael Hoelscher, Camilla Rothe

**Affiliations:** 1 Division of Infectious Diseases and Tropical Medicine, Medical Center of the University of Munich (LMU), Leopoldstr. 5, 80802 Munich, Germany; 2 German Center for Infection Research (DZIF), Munich, Germany

**Keywords:** *Plasmodium knowlesi*, Malaria, Thailand, Germany, Travel medicine

## Abstract

**Background:**

Thailand is a major destination for German travellers with more than 760,000 arrivals in 2015. At the same time, malaria is a concern in travel recommendations with regard to this destination. The World Malaria Report of 2016 mentions only *P. falciparum* and *P. vivax* as prevalent species for Thailand, however, *P. knowlesi* infections have been occasionally reported in Thailand. In German travellers, only five cases of *P. knowlesi* infections have been reported to date.

**Case presentation:**

A 45-year-old German male tourist travelled to Thailand from 25 December 2016 to 13 January 2017. On 14 January he developed fever with no other symptoms, and presented on 17 January at the Division for Tropical Medicine and Infectious Diseases in Munich, Germany.

Malaria was diagnosed, primarily based on a single parasite in the thin smear microscopy, while commercial rapid diagnostic testing remained negative. Only the result of a differential PCR assay revealed *P. knowlesi* infection.

**Conclusions:**

*P. knowlesi* has to be considered in travellers returning from Thailand. Cases may present with an extremely low parasitaemia. This is in contrast to the assumption that *P. knowlesi* was likely to cause high parasitaemia due to its short replication cycle.

## Background

Despite considerable reductions in morbidity, malaria remains to be a relevant public health concern for Southeast Asia. The WHO World Malaria Report 2016 estimates 52,000 malaria infections for the year 2015 for Thailand among a total population of 68.2 million [1]. At the same time, the region represents an important tourist travel destination. The total number of incoming tourists to Thailand has increased almost fivefold in the past 20 years, from 7 million per year in 1995 to 33 million in 2016 [2]. In 2015 more than 760,000 travellers from Germany visited Thailand, the majority for leisure purposes [3]. Thailand is in most parts considered a low risk destination concerning malaria infections in travellers, thus current preventive algorithms recommend exposure prophylaxis through use of insecticide treated bed nets and repellents for most major tourist destinations, without a general recommendation of antimalarial chemoprophylaxis. For longer trips that include stays in rural areas, German and Swiss guidelines, and the recent update of UK guidelines of 2017 recommend provision of an emergency standby medication [4, 5]. The CDC and the previous UK national guidelines advise taking antimalarial chemoprophylaxis for some rural areas, e.g. the forested areas bordering Cambodia, Myanmar and Laos [6, 7]. Recommended antimalarials for chemoprophylaxis are atovaquone/proguanil or doxycycline due to concerns over possible mefloquine resistance.

The incidence of malaria across all *Plasmodium* species in travellers to Thailand from 12 European countries and the USA has been reported by Behrens et al. at 0.60 cases per 100,000 visits for the year 2008 [8]. The species distribution for malaria infections in Thailand is reported by the World Malaria Report 2016 at 41.8% for *P. falciparum* and at 58.2% for *P. vivax* [1]. *Plasmodium knowlesi* infections are not reported at all in some publications, including the WHO world malaria report of 2016 [1], or as rare events in others. The occurrence of autochthonous infections in Thailand appears to be concentrated around the southern provinces [9–11].

*P. knowlesi* has been known as the “fifth malaria species” that naturally infects humans (recently there have been two distinct species identified in *P. ovale*, increasing the number of human-pathogenic species to six). In contrast to *P. falciparum*, *P. vivax* and *P. ovale* spp. it is mostly a zoonotic parasite that shows a high prevalence in macaques, in particular in Malaysia. The species *P. malariae* is known as an anthropozoonotic parasite. Different *Anopheles* species have been described as potential vectors, with divergent feeding affinities towards humans and non-human primates. First infections in humans were reported from Malaysia in 1965, and human infections have been reported as rare events ever since. However, in a retrospective study in Malaysia on samples of patients that had been diagnosed with *P. malariae* infections, actually 82% of infections were revealed by species-specific molecular analysis as having been caused by *P. knowlesi* [12] . Also problematic is the reported lack of reliability of common rapid diagnostic tests in detecting *P. knowlesi* infections [13]. *P. knowlesi* poses also challenges on malaria prevention efforts, as transmission appears to frequently occur outside homes, rendering bed net use futile [14].

In German travellers returning from Southeast Asia, *P. knowlesi* infections are analogously considered very rare events. In the available literature, there have been five cases reported in returning German travellers altogether, which have all been visitors to Thailand [15–19].

This article is presenting a further case of a *P. knowlesi* infection in a returning German traveller from Thailand, and highlights the difficulties in accomplishing the correct diagnosis. In addition, possible consequences for current travel recommendations are considered.

## Case presentation

### Clinical history and physical findings

**Fig. 1:**
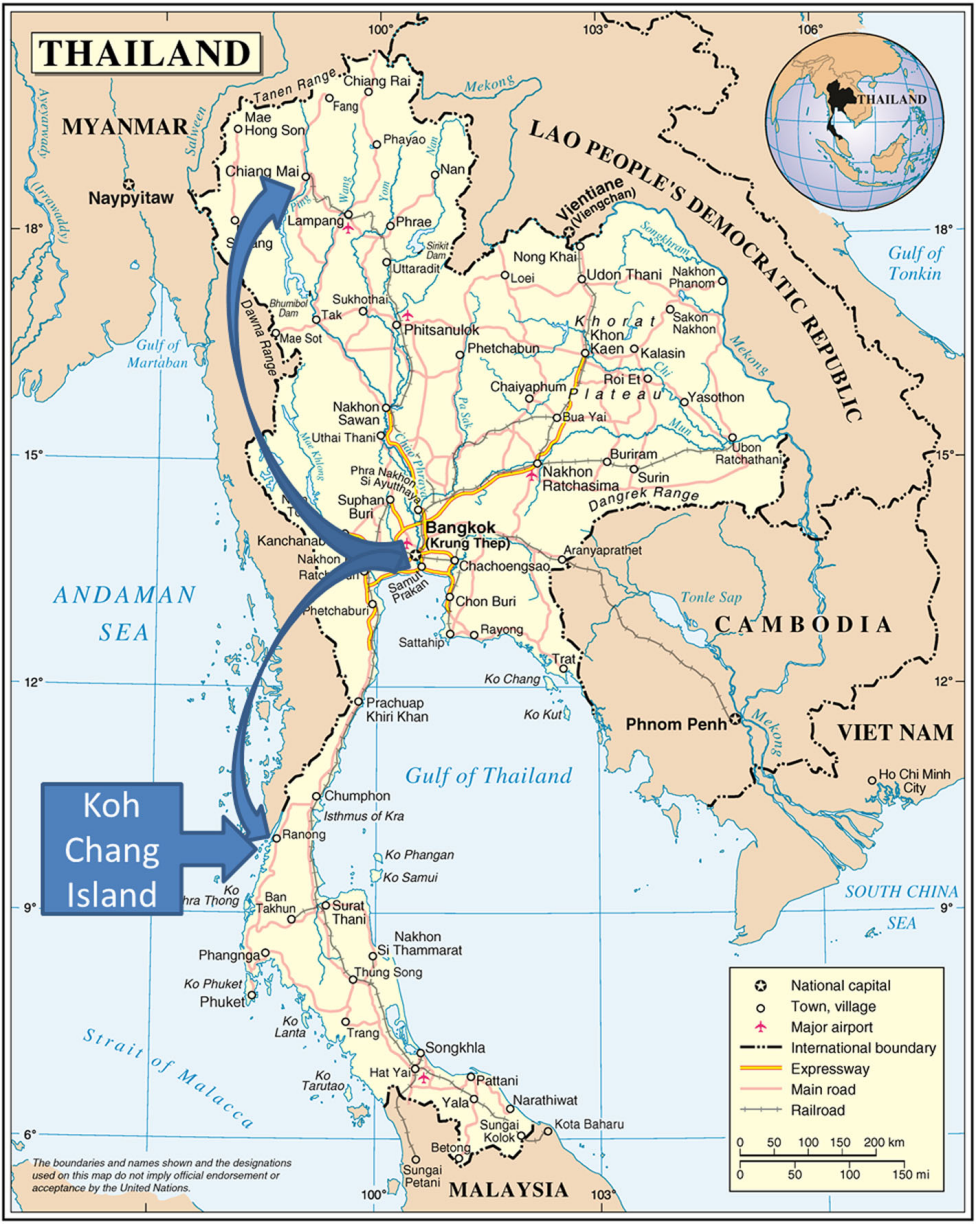
Map of Thailand with Itinerary. This map is based on a map by the Department of Field Support, Cartographic Section of the United Nations [30]. The map of Thailand shows the itinerary of the presented case

The patient, a 45-year-old male Caucasian German, born and residing in the German county of Bavaria, travelled to Thailand for holiday purposes repeatedly over the past years. The most recent journey started on 25 December 2016 with a flight from Munich, Germany to Muscat, Oman, where he had a 2-h stopover, before continuing to Bangkok, Thailand, where he arrived on 26 December. He spent the time from 26 December 2016 to 01 January 2017 in Chiang Mai in the north of Thailand where he visited the Doi Inthanon National Park. On 01 January he travelled to Bangkok where he stayed until 03 January, followed by a stay in Ranong city from 03 to 05 January. He then spent the time from 05 to 11 January on the island of Little Koh Chang in the Andaman Sea, which is part of Ranong Province. Via the city of Ranong he continued back to Bangkok where on 13 January he boarded a flight to Munich, Germany via Muscat, Oman (Fig. 1).

The patient travelled together with his partner who remained without health problems. He did not receive a specialized pre-travel consultation and did not take any antimalarial chemoprophylaxis, which is conforming to current German travel recommendations for Thailand. Regular use of repellents and of bed nets was reported. He did not reveal a medical history indicative of previous malaria infections.

### Clinical course

During the stay in Thailand the patient did not report any remarkable symptoms except painful and inflamed insect bites on two occasions while sleeping on the island of Koh Chang. On 14 January, the day after his return to Germany, he started to feel chills and developed fever up to 40 °C. He also developed profuse sweating, particularly at night time. Other symptoms such as arthralgias, myalgias or headaches were not present. He did not report any gastrointestinal, urinary tract or respiratory symptoms. After a brief consultation with the family physician in the patient’s rural area of residence in Bavaria on 16 January, he was referred to the Division of Infectious Diseases and Tropical Medicine (DIDTM) of the Medical Centre of the University of Munich (LMU) for further investigation.

Upon presentation, the patient’s general condition was only slightly reduced, temperature by tympanic measurement was 38.8 °C. The physical examination did not reveal any other remarkable findings. On abdominal ultrasound no pathological findings could be established, particularly the spleen appeared normal in size and ultrasound pattern.

### Laboratory diagnostics

Laboratory analyses were conducted in-house on whole blood and plasma samples upon attendance of the patient at DIDTM on day 4 after onset of symptoms. Automated full blood count was followed by visual differentiation of thin smears. As stipulated by the malaria emergency diagnostics protocol of DIDTM, screening for parasites was initiated by microscopical examination of thin smears on 100 fields at 400-fold magnification following Diff-Quik staining (Dade-Behring/Medion Diagnostics, Duedingen, Switzerland). Subsequently, Giemsa stained thick smears were examined. Furthermore, the rapid diagnostic tests BinaxNow Malaria (Alere, Scarborough, Maine, USA) for the detection of antigens of *P. falciparum* and/or *P. vivax*/*P. ovale* spp./*P. malariae*, Dengue Duo (SD Bioline, Yongin-si, Gyeonggi-do, Republic of Korea) for the detection of the non-structural protein 1 (NS1)/IgM/IgG and the Afinion C-reactive protein (CRP) test (Alere) were conducted. The *Plasmodium* genus-specific real-time PCR FTD Malaria (Mikrogen Diagnostik, Neuried, Germany) was carried out followed by the multiplex PCR FTD Malaria differentiation (Mikrogen) for the detection of *P. falciparum*, *P. vivax*, *P. ovale* spp. and *P. malariae*. In cases of positive amplification of *Plasmodium* genus-specific sequences and negative multiplex PCR FTD Malaria differentiation test results, the *P. knowlesi* nested PCR as first described by Imwong et al. based on a 410 base pair fragment of the species-specific *P. knowlesi* 18S rRNA gene is applied at DIDTM [20]. For immunodiagnosis an in-house immunofluorescence test employing *P. falciparum* and *P. vivax* antigens was conducted.

In addition, a full blood sample was sent to the national reference laboratory for PCR-based malaria differentiation at the Bernhard-Nocht-Institute for Tropical Medicine (BNITM), Hamburg, Germany.

## Results

As the automated blood count and the CRP rapid test results are conducted in-house at DIDTM, these results were available within the first half hour of presentation, and revealed a marked thrombocytopenia of 79/nl (normal range 140–360/nl) and an elevated CRP (later quantified at 10.6 mg/dl; normal range < 0,5 mg/dl). At the same time the Dengue rapid test and the malaria rapid diagnostic test were negative.

**Fig. 2:**
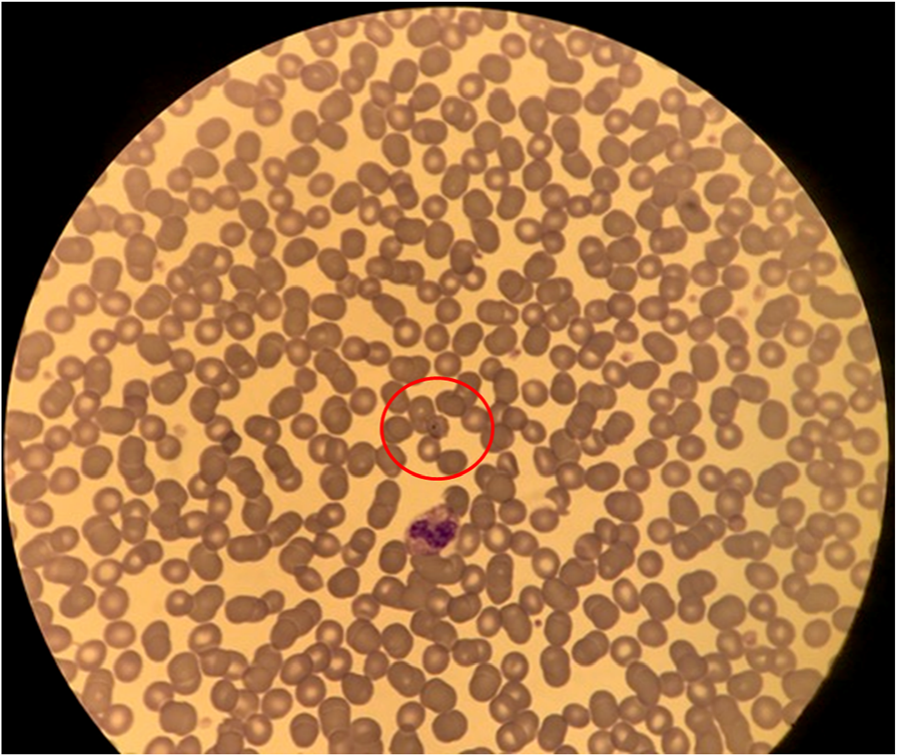
Photographic Image of a *P. knowlesi* Trophozoite. The figure shows a photographic image of a *P. knowlesi* trophozoite in a thin smear specimen of the reported patient at 400 x magnification

The thin blood smears revealed a *Plasmodium* infection at an extremely low parasitaemia. In the examination of 100 investigated fields at × 400 magnification only 1 parasite was detected. Consecutively the full thin smear slides were investigated, revealing a total of 3 parasites: one gametocyte and 2 trophozoites (parasite density 0.0002%) (Fig. 2).

The aspects of the identified gametocyte and the trophozoites rather resembled *P. vivax*. Examination of the thick smear again revealed only 1 structure suggesting a *Plasmodium* infection without allowing further differentiation. With this finding, treatment was initiated with a 3-day course of atovaquone 250 mg/ proguanil 100 mg, 4 tablets daily, as recommended by the national malaria guidelines of the Association of the Scientific Medical Societies in Germany [21]. Due to the only mildly reduced general condition of the patient the decision for outpatient management was taken. The same day the *Plasmodium* genus-specific PCR tested positive but the differential PCR revealed negative results for *P. falciparum*, *P. vivax, P. ovale and P. malariae*. Both the DIDTM and the national reference laboratory at BNITM returned a positive PCR result for *P. knowlesi*.

Fever and sweats settled the evening the first dose of atovaquone/ proguanil was given. The patient was seen again at DIDTM on 18 and 23 January, when repeated blood counts and smears were performed. Thrombocyte levels went down to 75/nl on 18 January, and returned to normal levels by 23 January. Blood smears on both follow-up days remained negative for parasites. The serology for *P. falciparum* and *P. vivax* remained negative. The patient improved without any signs of adverse treatment effects (Table 1).

**Table 1:** Timeline of Case History

Date	History
25 December 2016	Flight Munich, Germany to Bangkok, Thailand
05 January 2017	Passage to Koh Chang Island
11 to 13 January 2017	Return to Munich, Germany
14 January 2017	Onset of Symptoms
17 January 2017	Presentation at DIDTM, diagnosis of plasmodium infection, initiation of treatment
17 to 18 January 2017	Cessation of fever, clinical improvement
23 January 2017	Follow-up visit at DIDTM, normalization of thrombocyte count

## Discussion and conclusions

In the currently available literature, knowlesi malaria remains a rather rare event outside Malaysia, and particularly rare in travellers returning from Southeast Asia. Of note, the World Malaria Report 2016 does not state any *P. knowlesi* infection for Thailand, but only *P. falciparum* and *P. vivax* infections [1]. However, recent studies are indicating endemicity for *P. knowlesi* also in other Southeast Asian countries, such as Myanmar and Indonesia [22, 23].

Based on reported incubation periods for knowlesi malaria of about 9 to 12 days [24], it seems likely that the infection in this patient was acquired on the island of Little Koh Chang in the Andaman Sea, which is not to be confused with the much larger island of Koh Chang in the Gulf of Thailand. As macaques are the main reservoir of *P. knowlesi*, further studies are needed to assess the prevalence of the parasite in the monkey population on this island, which is a hideaway recommendation for tourists that intend to avoid the main crowds on the Thai islands on the gulf side.

As has been described in other publications, knowlesi malaria has to be suspected in situations where commercial rapid diagnostic tests reveal negative results in samples with positive blood smears [13, 17, 18]. The morphology of parasites in smears appears to be heterogeneous, and does not allow for a conclusive diagnosis [25]. Also, parasitaemia at presentation can be very low rendering parasite identification even more challenging, although potential hyperparasitaemia has been reported [25]. In cases where *Plasmodium*-species differentiation is hampered, it is recommended to do a differential PCR [26]. In the case presented here, the extremely low parasitaemia could even have led to a negative smear result in the first set of specimens. Only one gametocyte that was found in 100 visual fields of the blood smears led to the positive result of a plasmodium infection. The necessity of taking repetitive blood samples in the traveller with fever of unknown aetiology returning from malaria-endemic areas has to be kept in mind. In addition, an infection by *P. knowlesi* has to be suspected when the commercially available malaria PCR assays reveal a constellation of a positive genus specific sequence together with negative results for species specific sequences, as these usually do not comprise *P. knowlesi*.

As both artemisinine combination therapies [27] and atovaquone/ proguanil [28] seem to be effective in patients not showing severe illness with both *P. knowlesi* and *P. falciparum* infections, these should be considered as first line presumptive treatments where inconsistent laboratory results hamper an instant conclusive diagnosis. In our patient a rapid cessation of fever could be reached with atovaquone/ proguanil within 12 h of the first administered dose.

It is noteworthy that malaria prevention guidelines regarding travels to Thailand do differ between Germany, Switzerland, the UK and the USA. Both the German and the Swiss guidelines do not recommend antimalarial chemoprophylaxis for any region of Thailand, but recommend provision of standby medication for stays in the border regions particularly when outside reach of health care facilities [4, 5]. Contrary to that, the current US and the previous UK (until 2017) guidelines do recommend chemoprophylaxis with atovaquone/ proguanil or doxycycline for stays in border regions [6, 7], such as Ranong Province, bordering Myanmar, where our patient travelled to. The recent update of 2017 of the UK guidelines has now dropped the recommendation on chemoprophylaxis [29]. Adherence to the previous UK and current US guidelines would most likely have prevented this course of a *P. knowlesi* infection. Nevertheless, the authors are just able to present one case in this report, whereas recommendations for malaria prevention have to be based on risk modelling for geographic regions which are based on larger datasets. However, it has to be kept in mind that Thailand remains a favourite travel destination for German tourists, and diagnostic and treatment algorithms for febrile returning travellers have to take into consideration *P. knowlesi* infections. Diagnosis can be challenging due to ambiguous morphology of plasmodial parasites, very low levels of parasitaemia and negative results in commercially available rapid diagnostic tests. Treatment with atovaquone/ proguanil showed to be effective with rapid clinical improvement.

## Abbreviations

CRP: C-reactive protein
DIDTM: Division of Infectious Diseases and Tropical Medicine
PCR: Polymerase chain reaction
